# Global Solutions to Regional Challenges: Bridging the One Health Divide in the Caribbean

**DOI:** 10.1016/j.onehlt.2015.11.003

**Published:** 2015-12-02

**Authors:** Arve Lee Willingham, Luis Cruz-Martinez, Diana G. Scorpio, Christa A. Gallagher

**Affiliations:** a Ross University School of Veterinary Medicine, Basseterre, St. Kitts, West Indies

**Keywords:** One Health, Caribbean, Initiative, Framework, Trans-disciplinary

## Abstract

Ross University School of Veterinary Medicine, located on the Caribbean island of St. Kitts in the West Indies, hosted a multi-national, transdisciplinary One Health conference in St. Kitts and Nevis. Historically the many countries of the Caribbean carry a high burden of chronic and infectious disease and struggle with complex economic and developmental issues that continuously pressurize inhabitants and their natural environment. Considering these vast regional challenges, presentations covered diverse topics including community-based approaches for zoonotic disease control and prevention and mitigation of problems at the interface of wildlife, domestic animals, and humans. In two workshops the participants suggested a framework for practicing One Health in the Caribbean that emphasized capacity building and sustainability. Four structural components to the One Health paradigm were discussed including: identification of common problems, the necessity of comprehensive needs assessment, regional mobilization of resources, and building trust among all One Health stakeholders and the public.

In November 2014, Ross University School of Veterinary Medicine (RUSVM) hosted a multi-national conference entitled “Global Solutions to Regional Challenges: Bridging the One Health Divide”, in the Federation of St. Kitts and Nevis, West Indies. To convey the paradigm of One Health to all the conference participants, we endorsed the American Veterinary Medical Association’s definition of One Health which states that “One Health is the integrative effort of multiple disciplines working locally, nationally, and globally to attain optimal health for people, animals, and the environment” [1]. Furthermore, RUSVM, as an institutional member of the One Health Commission, advocates the Commission’s charter to “Educate and create networks to improve health outcomes and well-being of humans, animals and plants and to promote environmental resilience through a collaborative, global One Health approach” [2]. The purpose of the conference was to facilitate trans-disciplinary and cross-sectoral collaboration to enable the One Health model to be implemented in the Caribbean region. Ross University recognizes that in conjunction with its four research centers, it is effectively poised to promote and lead One Health efforts in this expanse of the western hemisphere. As the university is embedded in the Caribbean, it is intimately familiar with the particular pressures on this region which encompass limited resources, political and social issues revolving around emerging economies, health disparities, and the threat to the region’s terrestrial and marine biodiversity. Plenary, platform and poster presentations covered diverse topics, including but not limited to impact of zoonotic diseases on impoverished populations; community-based approaches for disease control and prevention; mitigation of problems at the interface of wildlife, domestic animals and humans; and translation of One Health concepts into practice in the developing world.

Oral/poster presentations were followed by workshops in which participants were allocated into focus groups to discuss specific topics. These groups included local, regional, and international participants from academia, health care researchers and practitioners, government officials and administrative personnel (see Fig. 1). The following paragraphs summarize the input from conference participants along with specific examples applicable to Caribbean One Health initiatives (see Fig. 2).

**Fig. 1:**
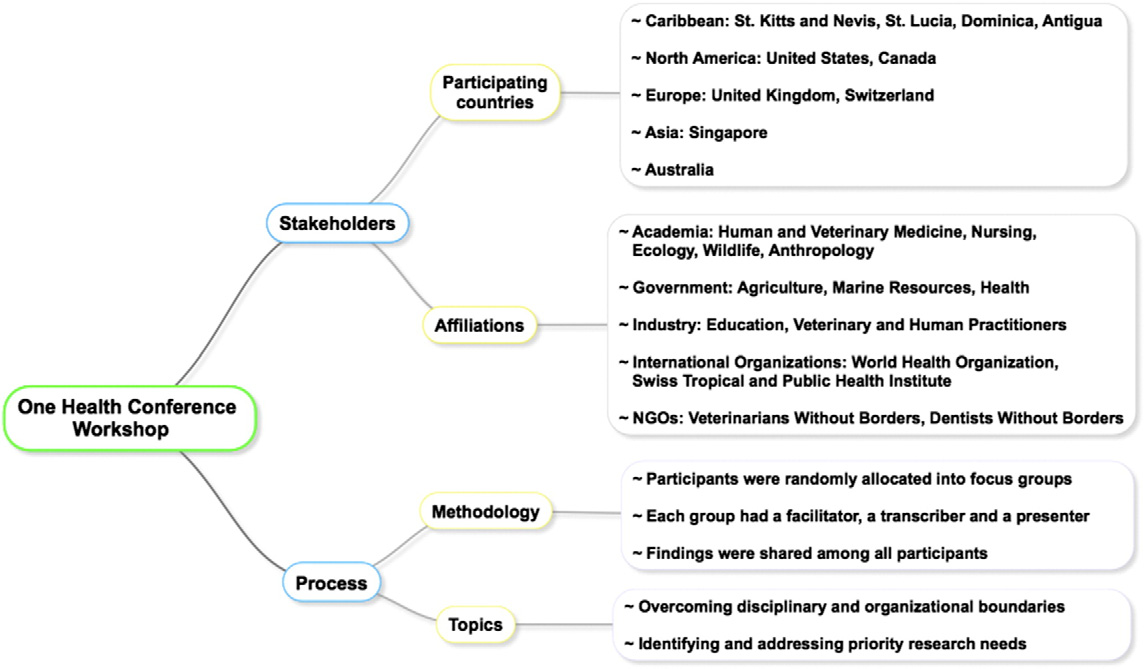
The conference attendants participated in a workshop to generate potential solutions to regional problems. There was a diverse group of stakeholders from multiple backgrounds, affiliations and nationalities; however, most participants belonged to academia from the United States and St. Kitts and Nevis and the majority were from the veterinary field.

**Fig. 2:**
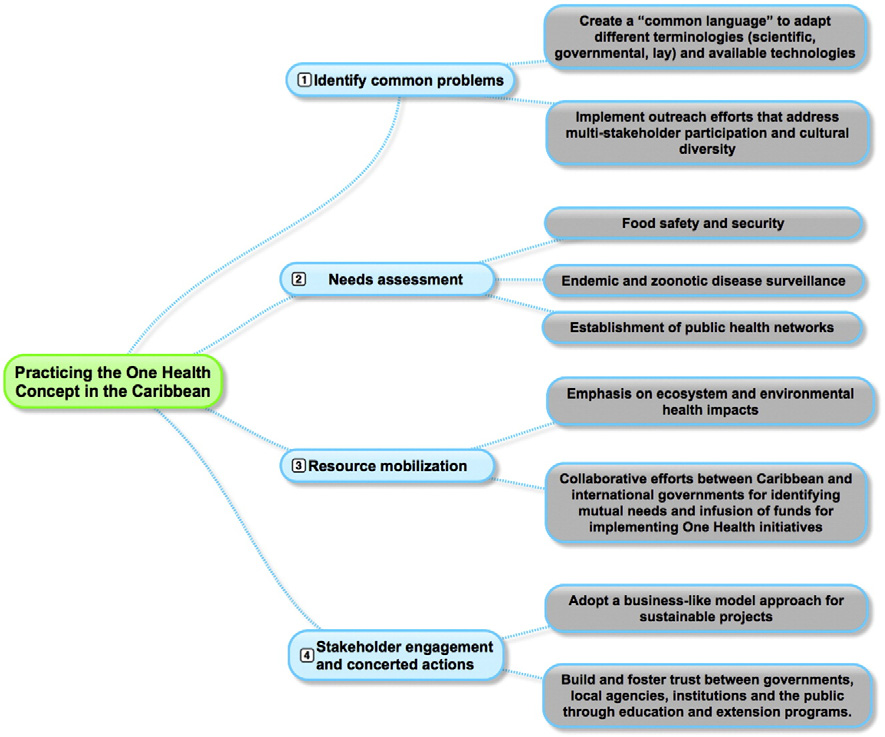
Framework and action plan to implement the One Health model in the Caribbean.

First, discussions focused on overcoming disciplinary and organizational boundaries for practicing One Health while highlighting effective communication, collaborations and coordination. A proposed first step is identification of a common problem or issue only solved through cross-sectoral collaboration. Once stakeholders are identified, interdisciplinary teams are formed. Communication across teams should involve a “common language” and require experts in communications, social science, information technology and administration to adapt different terminologies (scientific, governmental, and lay) into this “common language”. Main goals are to avoid jargon, and adapt language and technology to include diverse cultures. Along with communication and education, participants identified outreach efforts as a priority in One Health strategies. An immediate and urgent place to initiate One Health approaches is within higher education. Campus infrastructure and resources at most universities are not set up for collaborative work, and curricula often do not include development of soft skills needed to create One Health leaders. Beyond universities, this outreach should also target local NGOs and communities. An example of placing such an initiative into motion is our involvement in the “Monkey Task Force”, which involves cross-sectoral collaboration with RUSVM, Ministry of Agriculture, two local non-human primate biomedical research facilities, and local businesses. The aim of this task force has been to identify ways in which the African green monkey populations can be controlled, and how to minimize crop destruction, a significantly negative impact to local farmers. Progress has already been made to understand the extent of this issue and now collaborations are being formed to put multiple strategies into action, both locally and ultimately island-wide.

Second, establishing Caribbean One Health programs requires needs assessment. In most situations, this must occur following communications with stakeholders. Many times these needs must be realigned to attract funding agencies and their priorities, or global initiatives initiated in the Caribbean to serve as “model” or “pilot” interventions which demonstrate “proof of principle” for successful efforts. An important need identified involves securing resources for food safety and security via appropriate slaughterhouse practices, including public health-trained veterinarians and veterinary support staff. Other priority areas focused on implementation of surveillance systems for endemic and zoonotic diseases, and establishment of public health networks across the Caribbean, with recent chikungunya virus outbreaks as a relevant example.

Third, ecosystem and environmental health impacts were areas identified which require resource mobilization and intervention. The government of Taiwan has established a valuable presence in St. Kitts and across the Caribbean, and collaborative efforts with local governments are currently underway. Examples of multi-national government projects already completed with One Health implications in St. Kitts include an Eco-Park with greenhouses and demonstration farms for growing fruits and vegetables; a demonstration farm for educating farmers on compost techniques; and solar farms to provide continued clean energy, resulting in financial savings which can then be diverted to other community projects. This example is unique and provides a successful framework in identifying mutually beneficial needs and infusion of funds for implementing One Health initiatives. Workshop participants felt other agencies could develop similar programs, such as the Inter-American Institute for Cooperation on Agriculture (IICA), Food and Agriculture Organization of the United Nations (FAO), Ministries of Energy and Health for funding projects such as climactic influences on disease emergence, and lastly the Caribbean Development Bank (CDB), which can provide assistance in agricultural development. Financial commitments and volunteer opportunities from RUSVM, as well as other health-related regional institutes and interested NGOs such as Heifer International, could leverage support from larger funding agencies in driving One Health solutions. A measure taken by RUSVM to leverage some resources for continued One Health strategy implementation includes our recent membership in 2 influential organizations: the Association of Institutes of Tropical Veterinary Medicine and the Association of Marine Laboratories of the Caribbean. Member involvement in these organizations will help foster alliances and collaborations for stronger grant applications and membership opportunities and resources to implement important One Health strategies currently unfunded.

Fourth, stakeholder engagement and concerted actions was another major theme. Creating and fostering trust was noted as an essential foundation for collaborative work in Caribbean One Health. A common concern revealed a lack of communication and understanding between local governments and their constituents. This lack of engagement coupled with minimal government transparency is viewed as a major obstacle in establishing One Health initiatives. In addition, participants shared that trust between governments, local agencies, institutions and the public can be developed through educational extension programs. Examples such as community health fairs, agricultural extension, and primary school engagement were cited as example actions that would be viewed as culturally valuable and effective amongst Caribbean nationals. Concerted efforts to develop trust across stakeholders has included community efforts such as public radio talk shows on veterinary topics, RUSVM open house events for local communities, and graduate student projects which directly benefit community health problems in St. Kitts, with ultimate applicability to any Caribbean nation sharing similar One Health problems or issues. Also, with the implementation of the RUSVM One Health Master of Science degree, an online degree opportunity, the ability to reach world-wide impact has been realized, and students have the opportunity to work on problems in their own communities or they are encouraged to come to the Caribbean to study and solve many ongoing research problems. This has been a successful program thus far and has quite significant implications for Caribbean communities and direct implementation of One Health solutions.

As an example of extension to promote One Health, RUSVM is engaging the St. Kitts and Nevis Pig Producers’ Association to provide swine veterinary medical and husbandry education, as well as pork food industry management guidance. The goal of this project is to provide support to farmers to increase swine productivity and reduce disease burdens, thereby increasing national food security and sustainability. In conjunction with grassroots movements and political will, such programs provide evidence of success for sustaining One Health efforts.

To further grow stakeholder involvement in One Health, it was also suggested that a business-model approach be adopted. Interested stakeholders should develop business plans to support proposed One Health programs. Organizations should systematically identify potential collaborators and implement measurable One Health goals to build capacity in people and resources. As in any business model, One Health stakeholders should actively plan for funding resources to support their initiatives. It was the perception of participants that as a business is fiscally rewarded, so shall a One Health organization be rewarded following a solid plan with measurable achievements.

The conference demonstrated an effective way to enable knowledge sharing while promoting a trans-disciplinary One Health approach to solving relevant societal problems. Outcomes following group discussions provide the basis for a regional action plan to translate Caribbean One Health into an effort with positive impact on families, communities, populations and economies. The conference was successful in identifying achievable opportunities for transcending intra-disciplinary and organizational boundaries to foster a concerted, effective and sustainable effort to attain optimal health for people, animals, and the environment in a transformational way.
